# A Novel Anion Exchange Membrane for Bisulfite Anion Separation by Grafting a Quaternized Moiety through BPPO via Thermal-Induced Phase Separation

**DOI:** 10.3390/ijms21165782

**Published:** 2020-08-12

**Authors:** Md Mofasserul Alam, Yaoming Wang, Chenxiao Jiang, Tingting Xu, Yahua Liu, Tongwen Xu

**Affiliations:** CAS Key Laboratory of Soft Matter Chemistry, Collaborative Innovation Center of Chemistry for Energy Materials, School of Chemistry and Material Science, University of Science and Technology of China, Hefei 230026, China; mmalam@mail.ustc.edu.cn (M.M.A.); jcx11@ustc.edu.cn (C.J.); xutt49@mail.ustc.edu.cn (T.X.); liuyahua@mail.ustc.edu.cn (Y.L.)

**Keywords:** anion exchange membranes, BPPO, quaternized moiety, grafting, phase inversion, bisulfite, separation, electrodialysis

## Abstract

Ion-exchange membranes are the core elements for an electrodialysis (ED) separation process. Phase inversion is an effective method, particularly for commercial membrane production. It introduces two different mechanisms, i.e., thermal induced phase separation (TIPS) and diffusion induced phase separation (DIPS). In this study, anion exchange membranes (AEMs) were prepared by grafting a quaternized moiety (QM,2-[dimethylaminomethyl]naphthalen-1-ol) through brominated poly (2,6-dimethyl-1,4-phenylene oxide) (BPPO) via the TIPS method. Those membranes were applied for selective bisulfite (HSO_3_^−^) anion separation using ED. The membrane surface morphology was characterized by SEM, and the compositions were magnified using a high-resolution transmission electron microscope (HRTEM). Notably, the membranes showed excellent substance stability in an alkali medium and in grafting tests performed in a QM-soluble solvent. The ED experiment indicated that the as-prepared membrane exhibited better HSO_3_^−^ separation performance than the state-of-the-art commercial Neosepta AMX (ASTOM, Japan) membrane.

## 1. Introduction

Open-ended distinct characters of membranes have been considered and adopted in industrial uses. Ion-exchange membranes (IEMs) have significantly influenced the purification and separation process via electrodialysis (ED) [1] and other methods. A particular electric potential of the membrane solution interface has been declared to be required for co-ion repulsion through the membrane matrix (MM) in terms of the Donnan effect. As concerning this effect, as the external electrolyte concentration increases, the number of co-ions in the membrane phase increases [2]. To prepare novel IEMs with a simple technique (e.g., phase inversion [3]) and cheaply is notably arduous. The search for methods to develop a new type of IEM has intensified ED uses and increased the potential utilization. In general, covalent and ionic bonds are immobile relative to charged moieties on a polymer, which necessitates substituting moveable ions such as counter ions for increasing the hydrophilicity in the membrane configuration [4]. Hence, membrane affinity of required exchange depends on the hydrophobic zone, hydrophilic area of the water content, fixed ionic groups per unit weight, nature of the two solutions, polarization effect, etc. [5]. The hybrid IEMs are prepared through the evaporation of the solvent mixture to improve the membrane ion selectivity [6].

Since brominated poly (2,6-dimethyl-1,4-phenylene oxide) (BPPO) has access to benzyl halide groups, an alternative is possible regarding chloromethylation for subsequent quaternization. Moreover, BPPO polymer could be formed from another monomer through grafting without any catalysts [7]. Due to the qualities and compact-fit structures of the polymer backbone, the grafting technique is useful [8]. Grafting to, grafting through, and grafting from are conventional methods used for graft polymerization [9]. The permeability coefficient is inversely linked to the degree of cross-linking. It related to both the amine-functionality-of-weight percentage and the alkyl-site extent of the BPPO polymer. BPPO can have numerous types of modification such as alkylation, phosphorylation, bromination, and bromoalkylation [10]. Phase inversion, electro-spinning, stretching, and sintering are useful techniques for fabricating polymeric membranes [11]. Among them, phase inversion is a popular method for membrane production [12]. It includes the thermally induced phase separation (TIPS) and diffusion induced phase separation (DIPS) methods. In the TIPS method, the polymer is immersed in a poor solvent or solvent mixture and non-solvent at high temperatures. The membrane construction can be achieved by excluding the solvent via extraction.

Besides, naphthalene is a fused aromatic compound and has conjugated π-electrons. Among all the derivatives, 1-naphthol has a much higher melting point and comparatively low molar mass [13]. In previous work, the first commercial surface treatments for fluoropolymers were produced through a sodium–naphthalene complex in tetrahydrofuran (THF) [14]. The covalent immobilization of a crown ether moiety on the surface film and another quaternized moiety (QM) would be expected to increase the ion exchange capacity as required by membrane construction [15]. Moreover, one must consider the aromaticity effect of the ion separation technique induced by the conjugated π-electrons of the polymer MM [16]. Hence, the aromatic compound does not participate in binding but rather forms an aromatic core in the host [17]. Due to this perspective, we selected a 1-naphthol derivative aromatic alcohol (moiety) that is comparatively cheap and reactive in unique QM formation. Meanwhile, sodium bisulfite is known as a weak acidic salt. It used for soft reducing agents and de-coloration in purification. In wastewater processing, it often used to liquidate the effluent of the collecting water [18,19]. In this study, we used a weak sodium bisulfite acidic solution for bisulfite anion separation using ED for comparison with a commercial anion exchange membrane (Neosepta)(AMX) and desalination of bisulfate anions for further use, bearing in mind the stronger acid and product recyclable value. We sought to therefore widen the potential applicability.

In this innovation, a fresh derivative QM compound was used as functionalized using a 1-naphthol aromatic alcohol. Then, the moiety was quaternized over iodomethane. After that, BPPO was homogeneously dissolved in n-methyl-2-pyrrolidone (NMP), the solution was heated at 60 °C for about 4 h, and QM solution in NMP was added at 12 h, allowing grafting to occur through the quaternized membrane formed via the TIPS. Those membranes exhibiting grafting through anion exchange membranes (AEMs) were confirmed through the DIPS test in different solvents and with a high-resolution transmission electron microscope (HRTEM). Furthermore, uniquely developed membranes were favorable for bisulfite anion separation through ED.

## 2. Results and Discussion

### 2.1. Proton NMR and FTIR-ATR Spectrum

**Figure 1:**
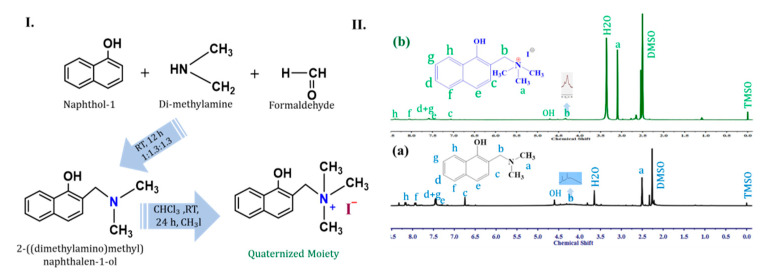
(**I**) Synthesis route for quaternized moiety (QM). (**II**) ^1^H NMR spectra of (**a**) 2-[dimethylaminomethyl]naphthalen-1-ol and (**b**) quaternized 2-[dimethylaminomethyl]naphthalen-1-ol.

The sound synthesis of the QM was carried out by following the procedure illustrated in Figure 1I. and confirmed through ^1^H NMR spectrum analysis (Bruker 400 MHz) as illustrated in Figure 1II [20]. In short, one proton peak was missing in the region 6.2 to 6.50 in the spectrum due to the aromatic proton of the moiety being functionalized. Hence, the N-CH_2_ peak at 4.20 to 4.40 showed a broad peak for the moiety, and a little sharp peak was observed for the QM independently. Herein, the QM had formed a methylene group that had an influence on the proton spectrum [21]. Moreover, the methylene protons showed an impact on the de-shielding chemical shifts at 3.10 to 3.20 due to the QM, while the existing moiety exhibited a chemical shift at 2.5. Meanwhile, commercial BPPO was confirmed by proton peaks in the regions 6.3–6.8, 4.2–4.4, and 2.0–2.4 individually, as addressed in Figure S2.

**Figure 2:**
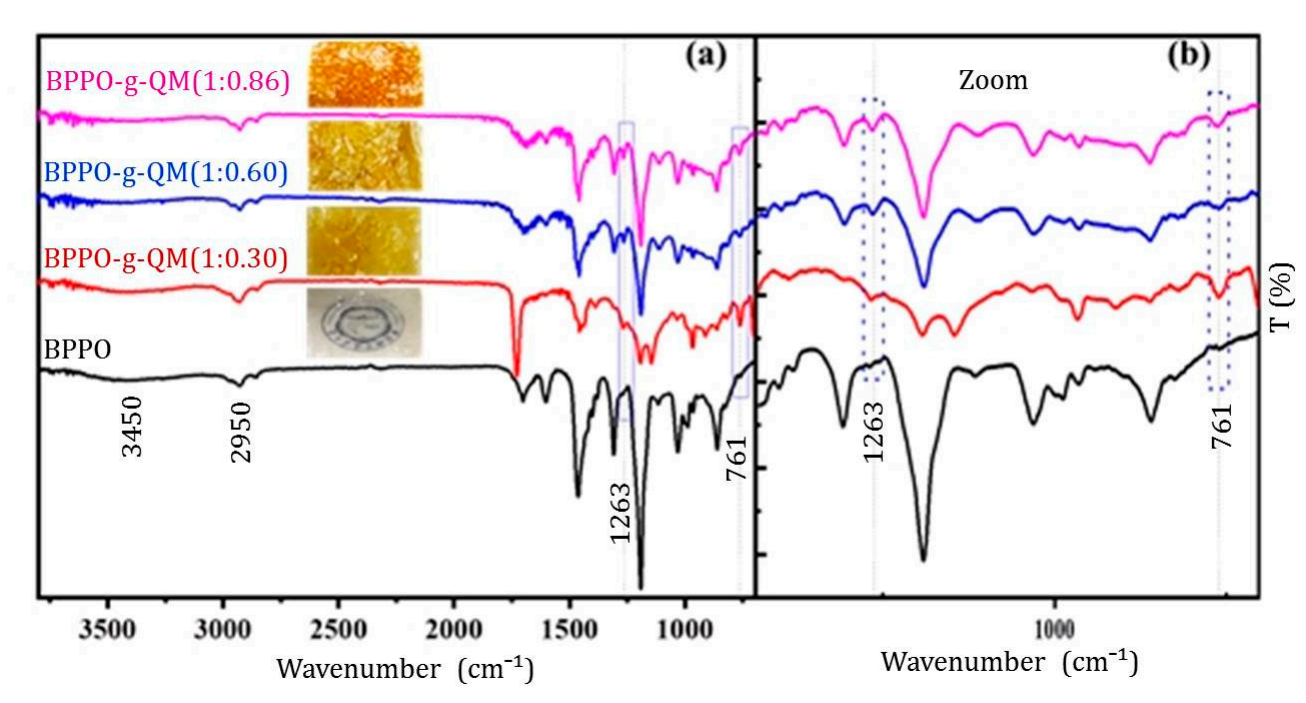
(**a**) FTIR-ATR spectra of (**a**) brominated poly (2,6-dimethyl-1,4-phenylene oxide) (BPPO) and QM membranes; (**b**) Spread wavenumber view.

The quaternized ammonium (QA) groups inside the membrane core developed during grafting were confirmed by the FTIR-ATR and results displayed in Figure 2. The commensurate study of the spectra of the BPPO, BPPO-g-QM (1:0.30), BPPO-g-QM (1:0.60), and BPPO-g-QM (1:0.86) membranes showed a medium absorption band around at 761 cm^−1^, which is ascribed to the C-N stretching vibration for the QA groups [7]. This band is unquenchable in the BPPO spectrum and manifested in Figure 2b. Most importantly, those outlines showed a preference for the characteristic peak of the QA groups. An absorption peak for the C-N stretching vibration of QA groups was observed at around 1263 cm^−1^, but it was absent in the BPPO. Meanwhile, an absorption peak at around 2950 cm^−1^ was facilitated by -CH_3_ functional groups. A broad absorption peak was detected at around 3450 cm^−1^ in the FTIR-ATR results, which was missing for BPPO. This defines the stretching vibration of 0-H bands due to the presence of water in the AEMs [22].

### 2.2. Tiny Characters

**Figure 3:**
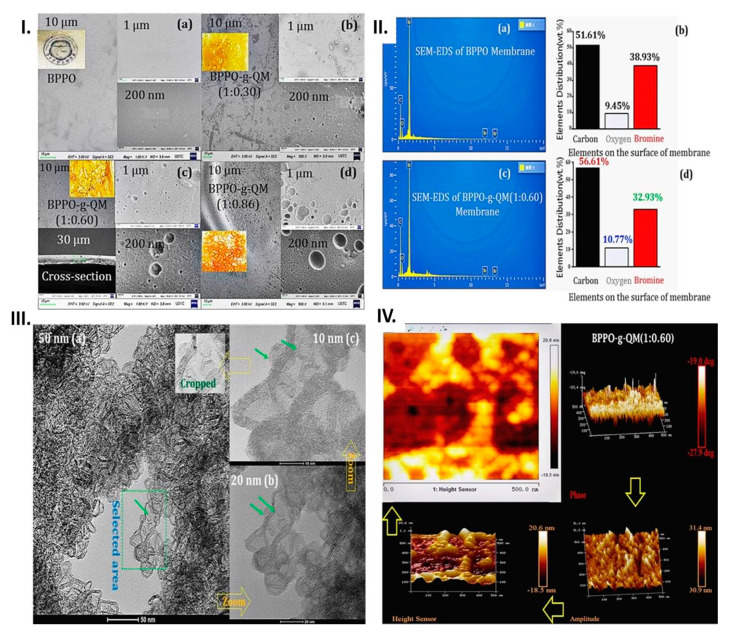
(**I**) SEM tiny images: (**a**) BPPO, (**b**) BPPO-g-QM (1:0.30), (**c**) BPPO-g-QM (1:0.60) and cross-section, and (**d**) BPPO-g-QM (1:0.86) membrane. (**II**) SEM-EDS surface element distribution. (**a**) BPPO and (**b**) analysis histograms, (**c**) BPPO-g-QM (1:0.60) membrane, and (**d**) analysis data. (**III**) High-resolution transmission electron microscope (HRTEM) images of BPPO-g-QM (1:0.60) in ethanol. (**IV**) AFM images of the surfaces of the BPPO-g-QM (1:0.60) membranes.

SEM was used to view the assembly of tiny microscopic phenomena. The surface images were distinguished from each other (see Figure 3I and, individually, Supplementary Figures S9–S12). Furthermore, SEM-EDS was performed for measuring the surface element distribution of the BPPO and BPPO-g-QM (1:0.60) membranes, as shown in Figure 3II. As concluded, the percentage of carbon and oxygen elements was increased; meanwhile, the bromine element content was decreased. The surface images showed that the enrichment effect of the QM content in the MM induced by the grafting through the TIPS occurred and that no holes or cracks were present. Scanning Probe Microscopy (SPM) was performed for the phase resolution, consisting of using a height sensor, determining the trapping roughness of the BPPO-g-QM (1:0.60) membrane, and producing a clear phase image, shown in Figure 3IV. The QM created an impression on the BPPO polymer’s morphology and increased the porosity, the roughness, the peak height, and a number of the peaks as well (see Figure S14).

Consequently, the prepared quaternized membranes were homogeneous in nature. For ED, a dense morphology of the MM is required, which can be observed from the microscopic images. Furthermore, to enhance the selectivity between hydrogen ions, hydroxide ions, and other anions in an acid–base interaction through the MM, the non-interconnected nanopores, and amphoteric topology could be considered. HRTEM was utilized to investigate the structural morphology of prepared BPPO-g-QM (1:0.60) quaternized AEMs. The images from the HRTEM are shown in Figure 3III, and the conformation and single caption are displayed in Figure S13. Herein, the images were amplified in the different ranges 50, 20, and 10 nm after dissolving in ethanol. It can be easily observed from the HRTEM images that the QM directly attached to the BPPO polymer due to grafting through copolymerization by the TIPS technique. Hence, HRTEM provided significant proof of the grafting in the fabrication of the MM.

### 2.3. Membrane Phenomenon

The ED process acts as effector for electro-potential. Annexing outcomes show that the ion exchange capacity (IECs) of prepared quaternized AEMs increase with an increase in QMs in the MM. Enriching the concentration of QMs in the MM was found to decrease them. In short, the hydrophilicity of the membrane might be improved. Moreover, the density of QMs in the MM affects the H_2_O absorption and thickness properties.

The transport number (TN) was found to be increased from the BPPO-g-QM (1:0.30) to the BPPO-g-QM (1:0.60) membrane by increasing the consistency of the QMs in the MM, except that BPPO-g-QM (1:0.86) showed a lower TN then BPPO-g-QM (1:0.60). Hence the diffusion of co-ions across the membrane is suppressed by rising quaternary ammonium groups (QAGs). The BPPO-g-QM (1:0.60) membrane achieved the highest TN (0.81) among all. This could be attributed to the highest content of QAGs through the optimum suppression of co-ion diffusion. Besides, the BPPO-g-QM (1:0.86) membrane obtained a lower TN due to the degree of substitution in the MM. The IEC, H_2_O absorption, hydrous angle, and chemical stability are essential properties (available in Table 1, and the details are in the Supplementary Materials) that have been demystified regarding the hydrophilicity of IEMs. As regarding experimental data of membrane phenomena, the BPPO-g-QM (1:0.60) membrane could be chosen for further treatment due to having the same QM symmetry as the MM.

### 2.4. Electrodialysis (ED)

**Figure 4:**
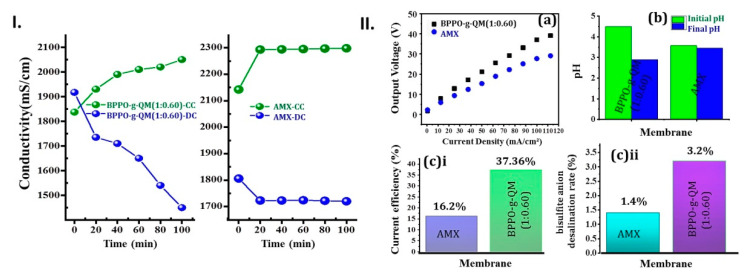
(**I**) Conductivity vs. Time plot in NaHSO_3_ solution at room temperature during ED. (**II**) (**a**) the potential difference of quaternized membrane BPPO-g-QM (1:0.60) and Neosepta (AMX) over stack during ED; (**b**) Changing pH of diluted cell; (**c**)i and (**c**)**ii**, the current efficiency and bisulfite anion desalination rates, respectively.

An ED test was performed at a laboratory scale to test the bisulfite anion separation performance of the BPPO-g-QM (1:0.60) membrane (for details, see the Supplementary Materials). The desalination performance was compared with that of a state-of-the-art commercial Neosepta AMX (Astom, Japan) [23,24,25]. The conductivity and potential difference of the NaHSO_3_ solution in dilute cell (DC) and concentration cell (CC) were changed in both cases, as shown in Figure 4I,II. The conductivity of DC could be interpreted for discussion; the anion and cation migrated through the AEM and cation exchange membrane (CEM) in the presence of an electric potential. The reduced concentration of ions in DC was much more eminent than the molar conductivity increase. Therefore, the net conductivity was decreased in DC. QAGs inside AEMs act identically to anions being transported through an anion exchange sequence. Besides, the remaining bromomethyl PPO group could be reacted with amine groups to form regular covalent bonds [26]. Furthermore, the pH value of DC throughout the initial and final time subsequently changed the pH value of the quaternized membrane instead of Neosepta AMX, as presented in Figure 4II.

Background chemistry would occur upon water dissociation (splitting) with AEMs that, through the weak primary group, resulted in reversible protonation and deprotonation [27]. The fixed functional group in the MM has a great influence on the intensity of water dissociation electrolytic mechanisms. Moreover, co-ions and counter-ions generate repulsive forces in the presence of AEMs due to water splitting in the ED [28]. The interplay among counter-ions and co-ions has a significant influence on the Coulombic force and hydration effect of the ions. Due to this effect, the SO_4_^2−^ anions are more electronegative than the HSO_3_^−^ anions, considering the consistency of the water dissociation therein [29]. Water dissociation reactions inside the membrane, including $\equiv N^{+},=N^{+},-N^{+},$ are more accelerated than in the membrane consisting of $-N^{+}$(CH_3_)_3_ groups. Such a characteristic might be correlated with the increasing ion-exchange content in the MM. However, the BPPO-g-QM (1:0.60) membrane was utilized in the ED system to separate HSO_3_^−^ anions from aqueous solution, showing a better desalination rate and current efficiency than Neosepta AMX.

## 3. Materials and Methods

### 3.1. Materials

1-naphthol (99%) was bought from Shanghai Aladdin Industrial Co. Ltd. (Shanghai China). Formaldehyde solution (37–40%), DMSO, NMP, hexane, ethyl acetate, ethanol absolute, methanol, iodomethane (CH_3_I), sodium hydrogen sulfite (NaHSO_3_), and trichloromethane (CHCl_3_) were purchased form Sinopharm Chemical Reagent Co. Ltd. (Shanghai China). Deionized water was used as necessary. Shandong Tianwei Membrane Co. Ltd. (Weifang China) provided the BPPO polymer. A 52% aryl degree of substitution was determined by ^1^H NMR (see Figure S2). The retail AMX and CMX membranes were used for comparing the results.

### 3.2. Methods

#### 3.2.1. Synthesized Moiety and Quaternization

A standard method was implemented to prepare the moiety as reported [30]. Formaldehyde (37 wt.% in H_2_O, 1.3 eq.) was added gently to the corresponding 1-naphthol and dimethylamine (33 wt.% in H_2_O, 1.3 eq.) at room temperature with continued stirring for 12 h. The product was extracted with CHCl_3_, dehydrated with anhydrous sodium sulfate, and concentrated in an exhausted drying oven. Finally, a hexane/AcOEt eluent (1:6) silica gel was used for the customized purification of the crude product by column chromatography. The QM was prepared through a single process shown in previous work [31]. The moiety and iodomethane (1:1.70) were dissolved in CHCl_3_ at room temperature for 48 h. After completion of the reaction, the precipitate was washed with ether (absolute) a minimum of five times and concentrated in a desiccator. Thereafter, the quaternary amine formation was verified by ^1^H NMR, as shown in Figure 1II.

#### 3.2.2. Thermal-Induced Phase Separation

**Figure 5:**
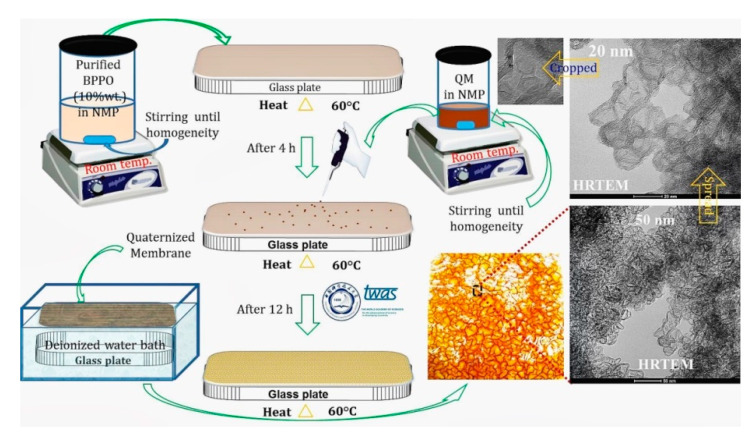
Scheme of BPPO-g-QM membrane grafting through the thermal induced phase separation (TIPS) technique and reaction.

**Figure 6:**
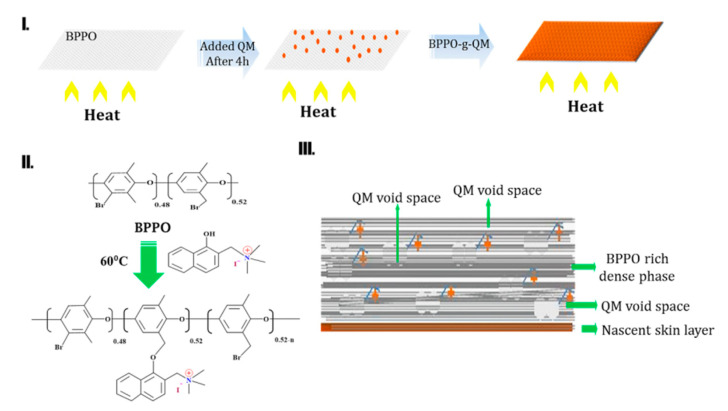
(**I**–**III**) Schematic of BPPO-g-QM membrane grafting through the TIPS technique and membrane conformation.

**Figure 7:**
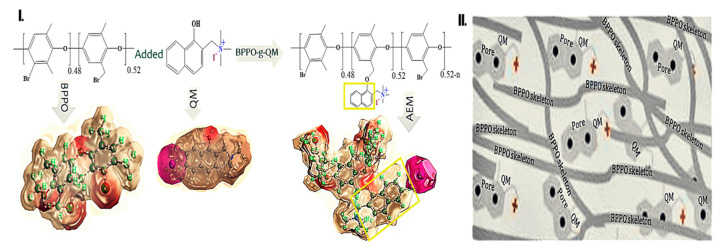
(**I**) Schematic representation as a ball-and-stick model of functionalization. (**II**) Schematic of membrane conformation grafting through QM.

For the polymeric membrane, the TIPS and DIPS techniques were used. The membrane morphology, nascent porosity, and surface formation are affected by the thermodynamics of the polymer solution [3]. TIPS is one of the leading procedures to afford microporous membranes. Herein, a de-mixing process occurs in phase inversion. The schematic invention of the BPPO-g-QM membrane is shown in Figure 5. The schematic membrane formation grafting through QMs is shown in Figure 6 and Figure 7II.

#### 3.2.3. Membrane Phalanx

The refined BPPO polymer was dissolved in NMP (10 wt.%) at ~ 5 °C to produce a solution. It was transferred onto the top of a horizontal glass plate, which was decorated with the metal plate with uniform heat through increasing the temperature up to 60 °C for ~ 4 h. Then, QM (1:0.30, 1:0.60 and 1:0.86) equivalent was added in 1 mL of NMP solvent before mixing gently to ensure analogy [32]. Therefore, the temperature of the thermo-plate was maintained at 60 °C for an extra 12 h (see Figure 6 and Figure 7). Later, the glass plate was dipped in water and the films were extracted.

#### 3.2.4. Membrane Grafting (DIPS Test)

DIPS modifies a polymer solution by three methods: immersion precipitation, vapor adsorption, and solvent evaporation [3]. Herein, the quaternized membrane (DIPS test) was investigated by controlling the weight variation and IEC value before and after immersion in ethanol and DMSO at room temperature (for details, see Supplementary Materials) [33]. Moreover, a selective BPPO-g-QM (1:0.60) membrane was used to inquire about the nanostructure construction through partial dissolution into ethanol by the low dose FE-HRTEM (FEI, Talos F200X) technique [34].

### 3.3. Characterization and ED

**Figure 8:**
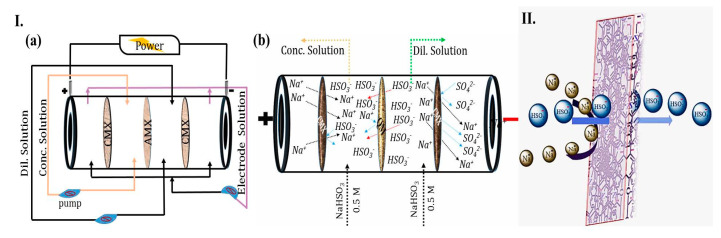
(**I**) (**a**) Schematic setup of ED stack separation and (**b**) mechanism. (**II**) 3D scheme for bisulfite anion separation.

The FTIR spectrum was tamed to analyze the AEMs, which was performed by applying an ATR technique (Vector 22, Bruker). The chemical stability was calculated by the deviation of the IEC and weight value before and after immersion in a 2 M NaOH solution at room temperature for a particular duration. The inner morphology was imaged by SEM (Sirion 500, FEI). Furthermore, the distribution of the elements on the surface SEM-(EDS) was studied. Additionally, AFM (Dimension Icon SPM) was used to observe the phase resolution and roughness. The thermic steadiness of the membrane was inspected with a TGA50H as regarding the temperature (25–800 °C) under continued N_2_ gas flow, with a heating rate of 10 °C/min. The mechanical properties were determined with an Instron Universal tester (DMA Q800) (for details, see Supplementary Materials). A well-known ED setup was functioned to test the separation efficiency of the developed membrane. The ED sequence is shown in Figure 8I and the Supplementary Materials.

## 4. Conclusions

An innovative AEM was successfully prepared with BPPO and QMs, applying the TIPS technique by grafting through copolymerization. The DIPS test was used to test the conduction of a QM-soluble solvent and showed excellent grafting. SEM images predicted the compact structures. Most importantly, HRTEM images showed the grafting configuration. The AEM seems to be reliable in terms of thermal and alkaline persistence. For separation of the ions, the prepared membrane was applied for bisulfite anion separation instated of AMX (Neosepta) and exhibited a better desalination rate. The principal bisulfite anion desalination rate (3.2%) obtained with the BPPO-g-QM (1:0.60) membrane was higher than that obtained with the commercial AMX (1.4%). The current efficiency (37.36%) achieved with the BPPO-g-QM (1:0.60) membrane was better than that with the commercial AMX (16.20%). However, the 1-naphthol base QM is a new functional material. So far, extending grafting through the 1-naphthol base with QMs in the MM has been demonstrated by HRTEM (showing a 2D clear conformation of QMs) construction, ultimately showing excellent performance of bisulfite anion separation in ED. Moreover, anion separation is the most complicated job due to different aspects. Consequently, particular anions, grafting structures, and strengthening of conjugated π-electrons through grafting should be analyzed in detail in the ion-separation field in our future work.

## Figures and Tables

**Table 1:** Experimental (physical, chemical, and electrical) data of anion exchange membranes (AEMs).

Membrane ^1^	AMX	BPPO-g-QM (1:0.30)	BPPO-g-QM (1:0.60)	BPPO-g-QM (1:0.86)
WU (%)	16	31.04	25.83	22.55
Thickness (µm)	134	58	110	100
IEC (mmol·g^−1^)	1.25	0.88	1.38	1.60
CA (±2)	-	75.08	81.10	82.60
AR (Ω·cm^2^)	2.35	8.19	3.08	2.14
TN	0.98	0.75	0.81	0.72
Ion Flux (mol·cm^−2^·s^−1^)	8.39 × 10^−12^	-	3.87 × 10^−11^	-
Current Efficiency (%)	16.20	-	37.36	-
Desalination rate (%)	1.40	-	3.4	-

^1^ All membranes were prepared with a constant ratio (BPPO equal to 0.505 mmol) of BPPO to QM (0.151, 0.306, and 0.437 mmol). Electrodialysis (ED) was performed for one hour for each case.

## Supplementary Materials

Supplementary Materials can be found at https://www.mdpi.com/1422-0067/21/16/5782/s1.

## Abbreviations

DD	Diffusion dialysis
ED	Electrodialysis
WU	Water uptake
TN	Transport number
AR	Area resistance
CC	Concentration cell
DC	Dilute cell
CA	Contact angle
DI	Deionized
AcOEt	Ethyl acetate
QM	Quaternized moiety
QA	Quaternized ammonium
MM	Membrane matrix
AEM	Anion exchange membrane
CEM	Cation exchange membrane
IEC	Ion exchange capacity
PEM	Polymeric exchangeable membranes
NMP	n-methyl-2-pyrrolidone
DMSO	Dimethyl sulfoxide
AMX	Anion exchange membrane (Neosepta)
CMX	Cation exchange membrane (Neosepta)
PPO	Poly (2,6-dimethyl-1,4-phenyleneoxide)
BPPO	Brominated poly (2,6-dimethyl-1,4-phenyleneoxide)
FTIR	Fourier transform infrared spectroscopy
ATR	Attenuated total reflection
NMR	Nuclear magnetic resonance
AFM	Atomic force microscope
FE-SEM	Field emission scanning electron microscope
FE-HRTEM	Field emission high-resolution transmission electron microscope
Moiety	2-[(dimethylamino)methylnaphthalen]-1-ol
QAGs	Quaternary ammonium groups
TIPS	Thermal induced phase separation
DIPS	Diffusion induced phase separation
BPPO-g-QM	Brominated poly (2,6-dimethyl-1,4-phenyleneoxide)-g-quaternized moiety
