# Supplementary material for: A Novel Anion Exchange Membrane for Bisulfite Anion Separation by Grafting a Quaternized Moiety through BPPO via Thermal-Induced Phase Separation
Source: Int J Mol Sci. 2020 Aug 12;21(16):5782. doi: 10.3390/ijms21165782 (PMC7460881; doi:10.3390/ijms21165782)
Supplement: Supplementary file 1 [file ijms-21-05782-s001.pdf]

International Journal of *Molecular Sciences*

MDPI

*Supplementary Materials*

# A novel anion exchange membrane for bisulfite anions separation by grafting a quaternized moiety through BPPO via TIPS

**Md Mofasserul Alam [1], Yaoming Wang [1] *, Chenxiao Jiang [1]*, Xu Ting Ting [1], Liu Yahua [1], Tongwen Xu ***

[1] CAS Key Laboratory of Soft Matter Chemistry, Collaborative Innovation Center of Chemistry for Energy Materials, School of Chemistry and Material Science, University of Science and Technology of China, Hefei 230026, PR China

* Correspondence: ymwong@ustc.edu.cn (Y.M. Wang); jcx11@ustc.edu.cn (C.X. Jiang); twxu@ustc.edu.cn (T.W. Xu); Tel.: (+86-551-63601587);

Received: date; Accepted: date; Published: date

## S1. Hydrous angle & $H_2O$ absorption

The immobile hydrous angle of the membrane surface was analyzed by a contact angle goniometer (Solon SL200B). The hydrophilicity of IEM was evaluated through calculating WU value. Herein, WU considers as $H_2O$ absorption phenomena. Firstly, the membrane was dried at 60°C for 24h in an exhaustion oven and impeccably weighed to confirm exact dried weight. Then the membrane was dipped into DI water for 48h at room temperature. The deviation weight of membrane before & after dried, $H_2O$ absorption value was aligned to relative weight achieved by per gram dry sample using equation[1].

$$\mathrm{WU}(\%) = \frac{W_{Wet}-W_{Dry}}{W_{Dry}} \times 100 \qquad (1)$$

Where, $W_{Dry}$ is denoted dry; and $W_{Wet}$ is denoted wet weight of the membrane sample respectively.

## S2. Area Resistance of the surface

A commercial cell-model from Hefei Chemjoy Polymer Material Co., Ltd. was utilized to evaluate the surface area resistance shown in Figure S1. During the experimentation, $Na_2SO_4$ solution (0.3 $molL^{-1}$) was submerged to the electrolyte for electrode cells, and NaCl solution (0.5 $molL^{-1}$) was circled to the intermediate cells. Direct current equipment (HSPY-60-05) was used at a constant (I = 0.04A). Furthermore, a multi-meter (VC890C+) was utilized for measuring the potential deviation. The area resistance of the surface was calculated according to the below equation [2].

$$R = \frac{U-U_o}{I} \times S \qquad (2)$$

Where, R is signified the area resistance of the surface; S is implied the actual surface area (7.065 $cm^2$); U is denoted the voltage with membrane; and $U_0$ is denoted the voltage without membrane holder respectively.

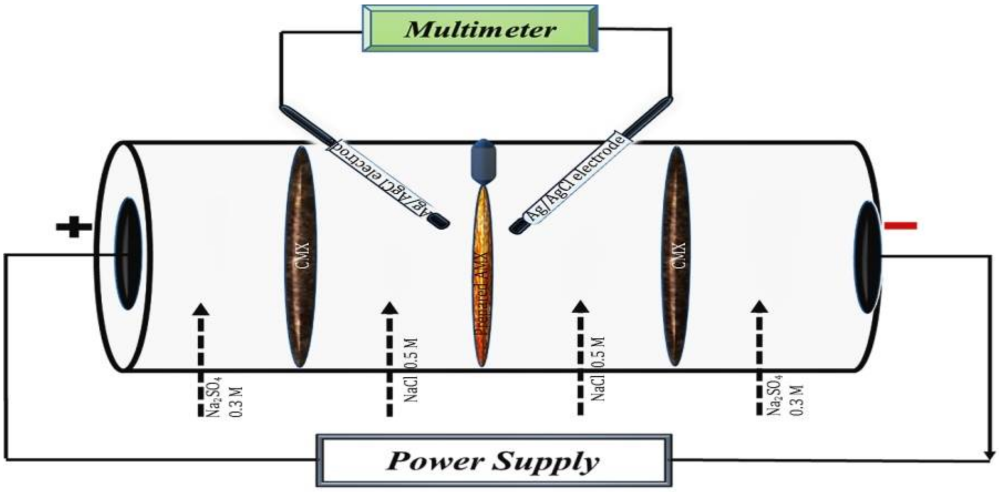


**Figure S1.** Schematic setup of membrane surface area resistance, which was filled with supporting electrolyte solution (0.3 M Na2SO4) and feed solution (0.5 M NaCl) as per shown the direction flow through the above diagram

## S3. Ion Transport Number

A block-cell formed by the Perspex sheet was used to measure the potential variation. It had two chambers divided by the membrane area around 7.07cm$^2$. The potential deviation was measured by keeping the salt concentration inside the lower chamber 0.01M NaCl and higher chamber 0.05 M NaCl solution respectively. The cell potential difference (Em) was calculated by utilizing a multi-meter (**VC890C+**). Ag/AgCl was used to reference electrode. The transport number ($t_i$) was measured through connecting qualified Nernst equation [2].

$$E_m = \frac{RT}{nF}(2t_i - 1)\ln\left(\frac{a_1}{a_2}\right) \quad (3)$$

where, R is specified universal gas constant (8.314 J/K.mol); F is defined the Faraday constant (96,487C/mol); T is named the absolute temperature (K); $a_1$ & $a_2$ are denoted mean activities of electrolyte solutions; and n is counter-ion respectively.

## S4. Membrane surface Ion Exchange Capacity (IEC)

The prepared quaternized membrane prolific IEC was measured through Mohr method as reported [3]. Firstly, sample was counterbalanced in 1M NaCl solution for two days regarding the formation of $Cl^-$ ions. Secondly, membrane was washed by DI water to mop an excessive quantity of NaCl from membrane surfaces. Thirdly, membrane was immersed in 0.5M $Na_2SO_4$ solution till two days for replacing immobilized $Cl^-$ ions instated of $SO_4^{2-}$ ions. The amount of releasing $Cl^-$ ions measured through gravimetric titration method by 0.05M $AgNO_3$ solution. Herein, $K_2CrO_4$ was an indicator.

$$\text{IEC}\ (mmolg^{-1}) = \frac{V_{AgNO_3} \times C_{AgNO_3}}{M_{dry}} \quad (4)$$

Where $M_{dry}$ signifying the dried weight of the membrane; $V_{AgNO_3}$ is represented the titrated value; and $C_{AgNO_3}$ is denoted the concentration of $AgNO_3$ solution respectively.

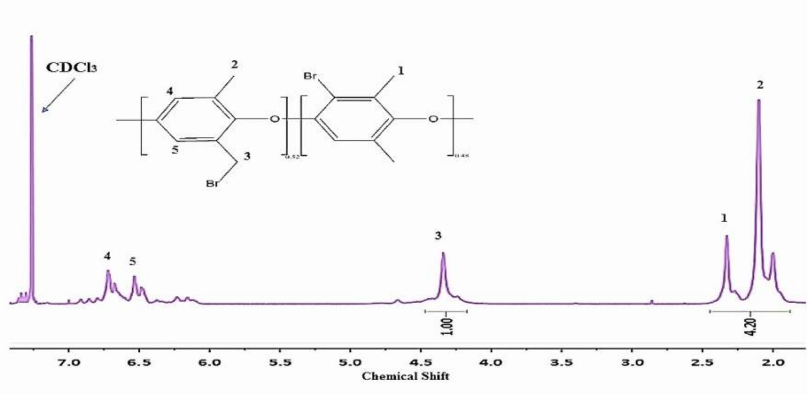


**Figure S2.** 1H NMR of Purified BPPO view in CHCl3 solvent

**S5. Membrane Grafting Test (DIPS Test)**

The diffusion is influenced by contact time, temperature, polymeric unit weight, and physical structure. Besides, the polarity would be increased by the adhesion technique while considering the consequences. Therefore, an electron-withdrawing group directly attached to the backbone and allowed leaving for the formation. So capping the β-hydrogen's into long alkyl chains can inhibit the Hofmann elimination reactions beyond of high electron density [4]. In this inquiry, we were performed selective BPPO-g-QM (1:0.60) membrane for the DIFS test to view the grafting performance into BPPO polymer using Ethanol & DMSO as considering the solubility of the QM, shown details in **Figure S3** & **Figure S4** respectively. In both cases, the IEC and Weight values were remained constant at nearly 200 h, except had a little change in their appearance, but after changed at around (210-280) h and then unchanged up to 485 h.

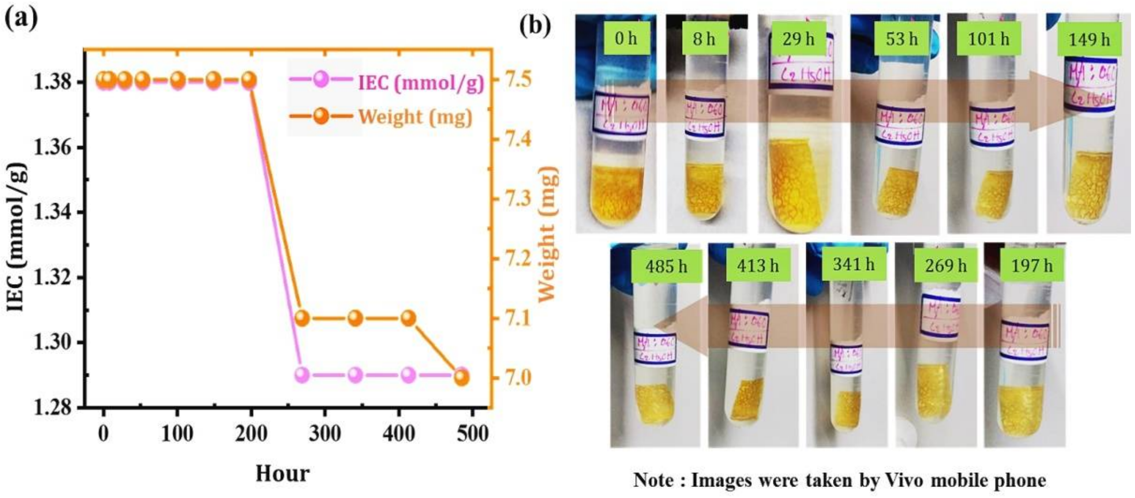


**Figure S3.** The membrane grafting (DIPS Test) in Ethanol for selective BPPO-g-QM (1:0.60) at room temperature of a specific duration: I. IEC (mmolg-1) & Weight (mg) value; II. Immobile images were taken in Ethanol

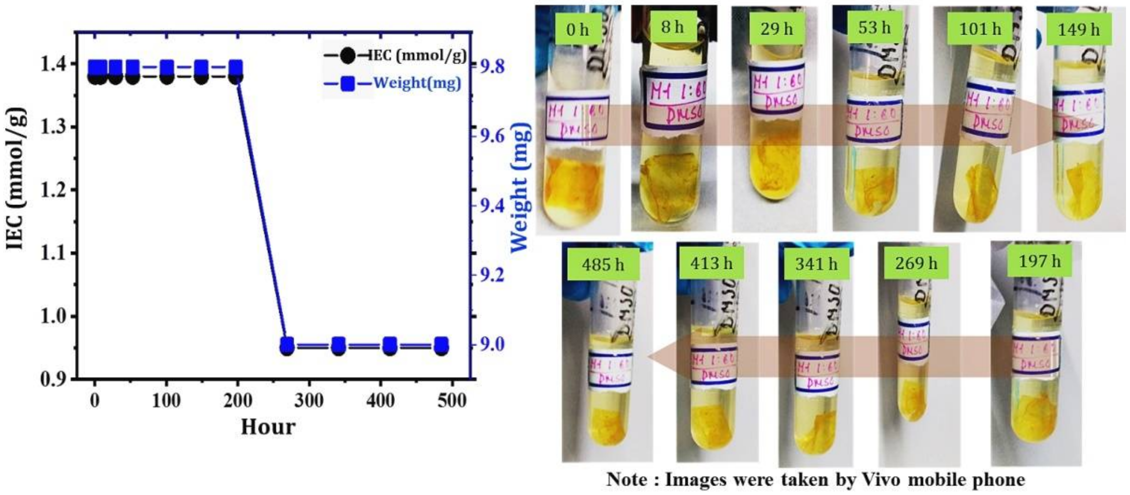


**Figure S4.** The membrane grafting (DIPS Test) in DMSO for selective BPPO-g-QM (1:0.60) at room temperature of a specific duration: I. IEC (mmolg-1) & Weight (mg) value; II. Immobile images were taken in DMSO

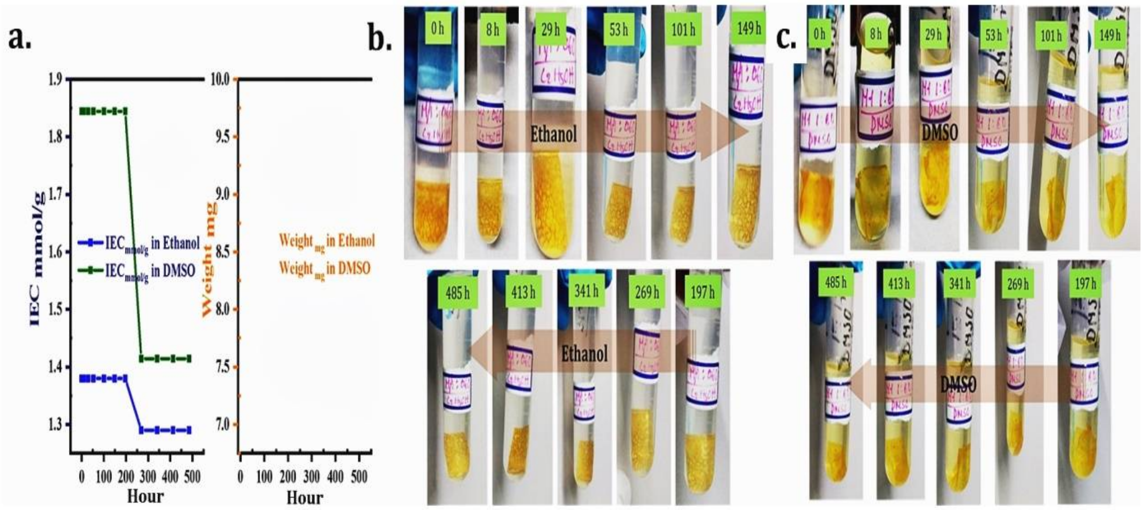


**Figure S5.** a. comparative studies of IEC (mmolg-1) & Weight (mg) value of (DIPS Test); b. Images in DMSO at room temperature of a specific duration for BPPO-g-QM (1:0.60) membrane ,and c. Ethanol at room temperature of a specific duration for BPPO-g-QM (1:0.60) membrane respectively.

Using Solvent Adhesion, some materials or synthetic compounds would be joined through the DIPS technique[5]. That could be transpired either in the mobile phase nor soluble in each other. As the diffusion theory, the polymeric materials incorporated through the interpenetration of chains due to adhesion. In this query, both QM and BPPO polymer were dissolved in DMSO; while the QM is dissolved in ethanol with excellent solubility (see **Figure S5**). It is concluded that QM could be attached to the BPPO polymer backbone as interpreting grafting through the TIPS[6]. As noted, it is high basicity and hydroxide ion likely to attack polymer MM under thermal conditions.

## S6. $H_2O$ absorption, hydrous angle and Chemical stability

$H_2O$ absorption (WU) is a primary parameter of IEMs and has created an effect on the separation so far the mechanical phenomenon follow through former work. For prepared AEMs, it seems to be improved in the MM. Most importantly, the density of QM in the MM was affected the $H_2O$ absorption and thickness properties. The surface CA (hydrous angle) of the prepared quaternized membrane was improved with the decreasing consistency of QM in the MM, as displayed in **Figure S6.I**. Amongst all, the BPPO-g-QM(1:0.60) membrane was used to diagnosis for chemical persistence. The membrane was dipped into the NaOH (2M) solution. The evaluation of alkaline

stability relates to the changed IEC(mmolg$^{-1}$) and weight (mg) value of membranes. The resultant data presented in **Figure S6.II** and performed membrane images into a 2M NaOH solution displayed in **Figure S6.II**. As for more extended stability of prepared quaternized membrane under actively alkaline conditions are best used for the electrochemical application [4]. Moreover, highly alkaline stabilizes membrane should be stable at a pH range 0 to 14 in oxidizing agents was reported [7].

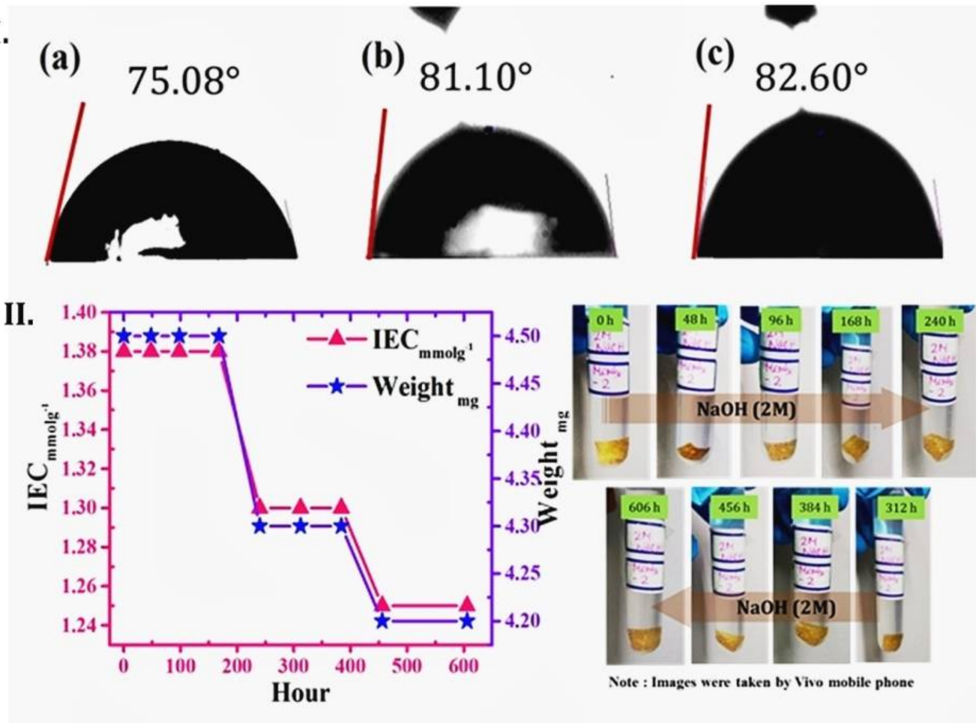


**Figure S6.** I. Stationary CA (hydrous angle) of the membrane (a) BPPO-g-QM(1:0.30), (b) BPPO-g-QM(1:0.60), and (c) BPPO-g-QM(1:0.86) respectively; II. Alkaline stability of prepared BPPO-g-QM(1:0.60) membrane in 2M NaOH solution at room temperature for a specific time **duration.**

**S.7 Electrodialysis (ED)**

ED experiment was carried out with the feed solution 0.5M $NaHSO_3$ owing to DC through a regular flow rate at 60 mL/min by applied low current density 20 mA/cm$^2$. The developed quaternized AEM used cross-sectional area about to 2.00 cm$^2$ for the ED cell. Two electrode cells were filled over 0.3M $Na_2SO_4$ solutions, while the conc. cell and dilute cell were fed with 0.5M $NaHSO_3$ solution. Each cell was diffused for 30 minutes to reduce the possible bubbles and conducted under a uniform current density upto 20 mA/cm$^2$. The conductivity of $NaHSO_3$ solution inside conc. cell, dilute cell and potential variation into the ED stack were remarked per 20min sequentially. The separation of ED workout was accelerated through prepared quaternized AEM & Neosepta AMX. Moreover, the results were examined in terms of desalination rate [8], current efficiency [9] and ion flux [10] and calculated by the below equations 5, 6, 7 [Herein, ICP OES 7300DV used to measure the concentration of ions].

$$\text{Desalination Rate (\%)} = \frac{C_0 - C}{C_0} \times 100 \quad (5)$$

Where $C_0$ is denoted initial; and $C$ is denoted final salt concentration (mg/L) respectively

$$\text{Current efficiency (\%)} = \frac{(C_t - C_0)\,V\,F}{It} \times 100 \quad (6)$$

Where $C_t$ is signified initial; and $C_0$ is signified final concentrations of bisulfite anion in molL$^{-1}$ at time t and 0 respectively; V is represented the volume of the diluted chamber; F is represented the Faraday constant (96485 C mol$^{-1}$) and I (A) is signified applied current.

$$\text{Ion flux (mol cm}^{-2}\text{s}^{-1}) = \frac{(C_t - C_0)\,V}{A_m t} \quad (7)$$

where $C_t$ is represented initial; and $C_0$ is denoted final concentration of bisulfite anion in molL$^{-1}$ at time t and 0 respectively; V is denoted the volume of the diluted chamber; and $A_m$ is denoted the active surface area of the membranes.

## S.8 Thermal stability

In the TG analysis, the degradation of the QAGs attributed to a weight of loss around 245°C. In short, the addition of the gap between the QAGs and the polymer backbone are increased the thermal stability of the polymer matrix. Two critical aspects, i.e. Td & IDT, were calculated and marked in **Figure S7.** The IDT values of prepared membrane was given in the range of (180-190)°C, whereas Td values consisting as the limit of (229-245)°C, to compare with IDT and Td values of BPPO blank membrane were shown 184°C and 210°C respectively. The better thermal stability facts get from IDT & Td values by the degradation of QAGs of these AEMs. Therefore, resultant of the prepared AMEs seems to excellent thermal stability in character.

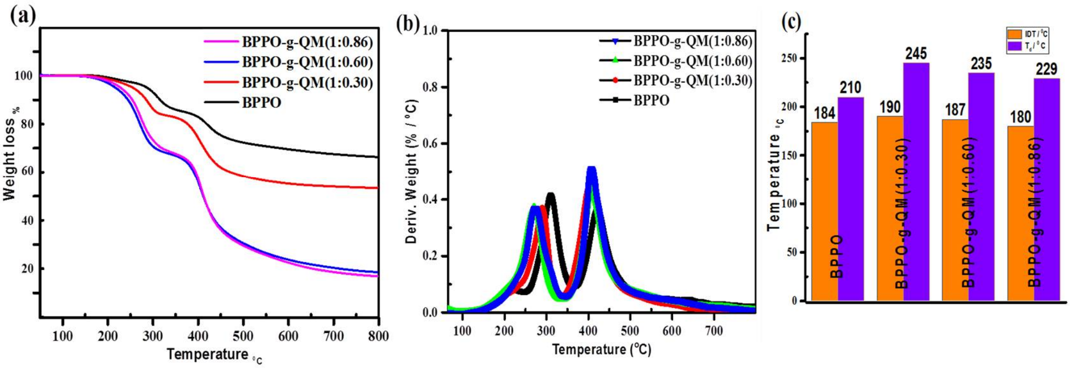


**Figure S7.** TG Analysis curve of the prepared Membranes: (a) Weight loss (%); (b) Derivative weight (%) per degree Celsius; (c) data analysis of prepared quaternized membranes respectively.

## S.9 Mechanical Stability

The mechanical features of quaternized AEMs as considering TS and Eb were explored in wet condition. The TS of qauternized BPPO-g-QM (1:0.60) membrane was found to decrease with enhancing the density of QM in the MM exhibited in Figure S8. Besides, other prepared membranes shown lower value due to the concentration ratio exhibited grafting through BPPO copolymerization. Meanwhile, the static force (N) of all quaternized membranes increased through improving the absorption of QM in the MM. As resultant excellent TS conquered from the quaternized membranes. It could be seen that the quaternized membrane BPPO-g-QM (1:0.60) have the highest TS and lowest $E_b$ value, representing the higher flexibility in their construction form. The higher degree of BPPO polymer with QM concentration will be improved the mechanical strength of the prepared membranes, while the higher density of QM in BPPO had led to a low TS value intending low chemical resistance as well. The TS of AEMs steadily was decreased from BPPO-g-QM (1:0.30) to BPPO-g-QM (1:0.60) and increased the IEC (appearing to form the incorporation of ion-exchange groups into the initially stable polymer string channels) [11]. However, the prepared AEMs possess enough mechanical stability and sufficient for ED application since considering their experimental values.

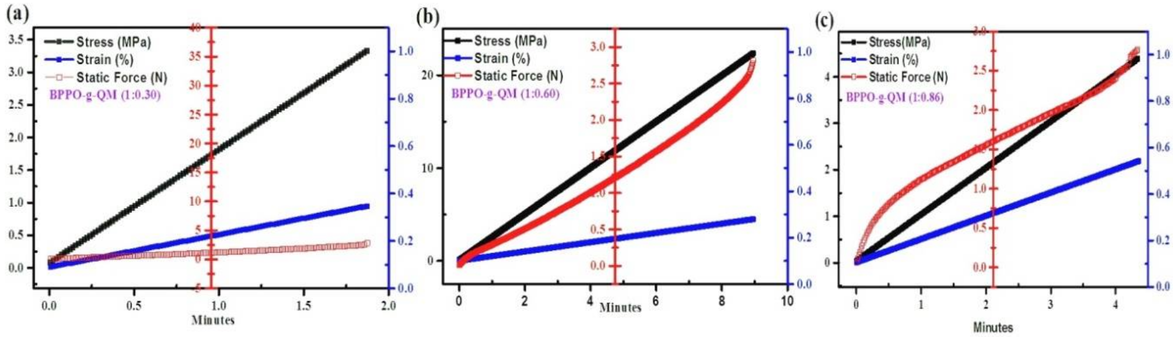


**Figure S8.** DM Analysis data of the prepared quaternized Membranes: (a) BPPO-g-QM (1:0.30); (b) BPPO-g-QM (1:0.60); and (c) BPPO-g-QM (1:0.60) respectively.

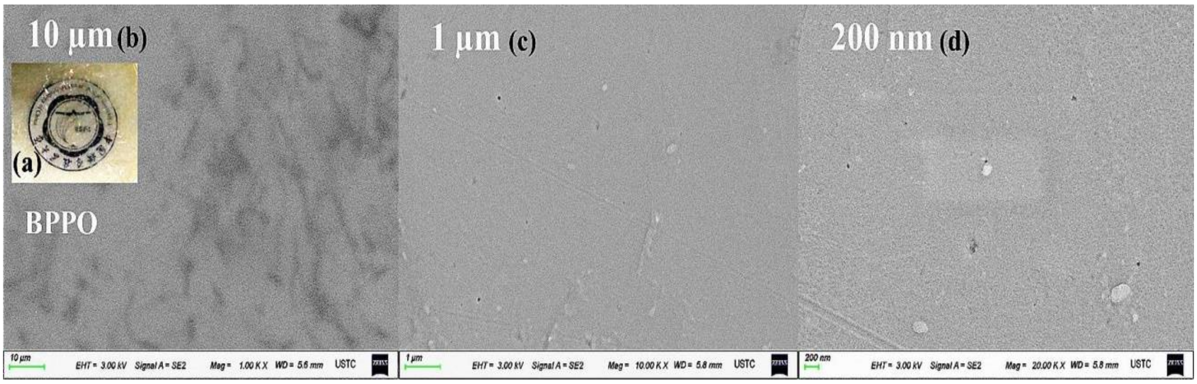


**Figure S9.** (a) Prepared BPPO membrane and Field Emission Scanning Electron Microscope (FE-SEM, Sirion500, FEI Company, USA) as consisting magnification of: (b) 10 µm (c) 1 µm, and (d) 200 nm respectively.

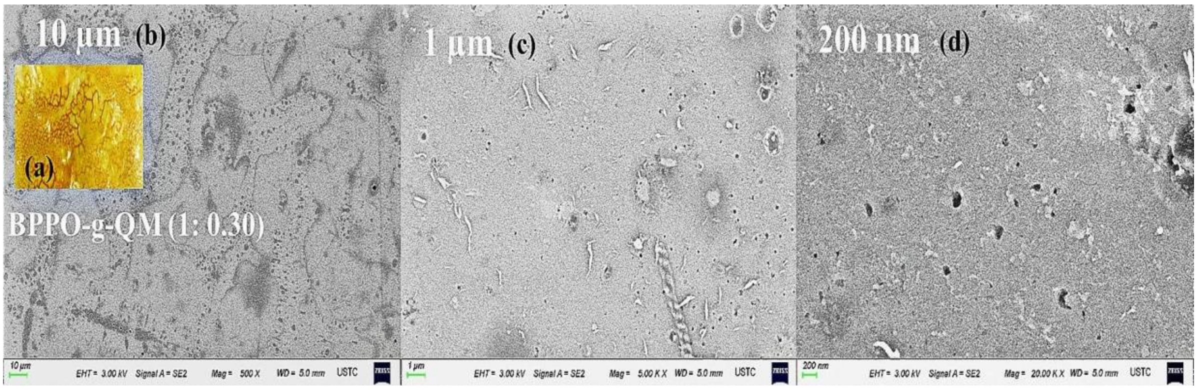


**Figure S10.** (a) Prepared BPPO-g-QM (1:0.30) membrane and Field Emission Scanning Electron Microscope (FE-SEM, Sirion500, FEI Company, USA) as consisting magnification of: (b) 10 µm (c) 1 µm, and (d) 200 nm respectively.

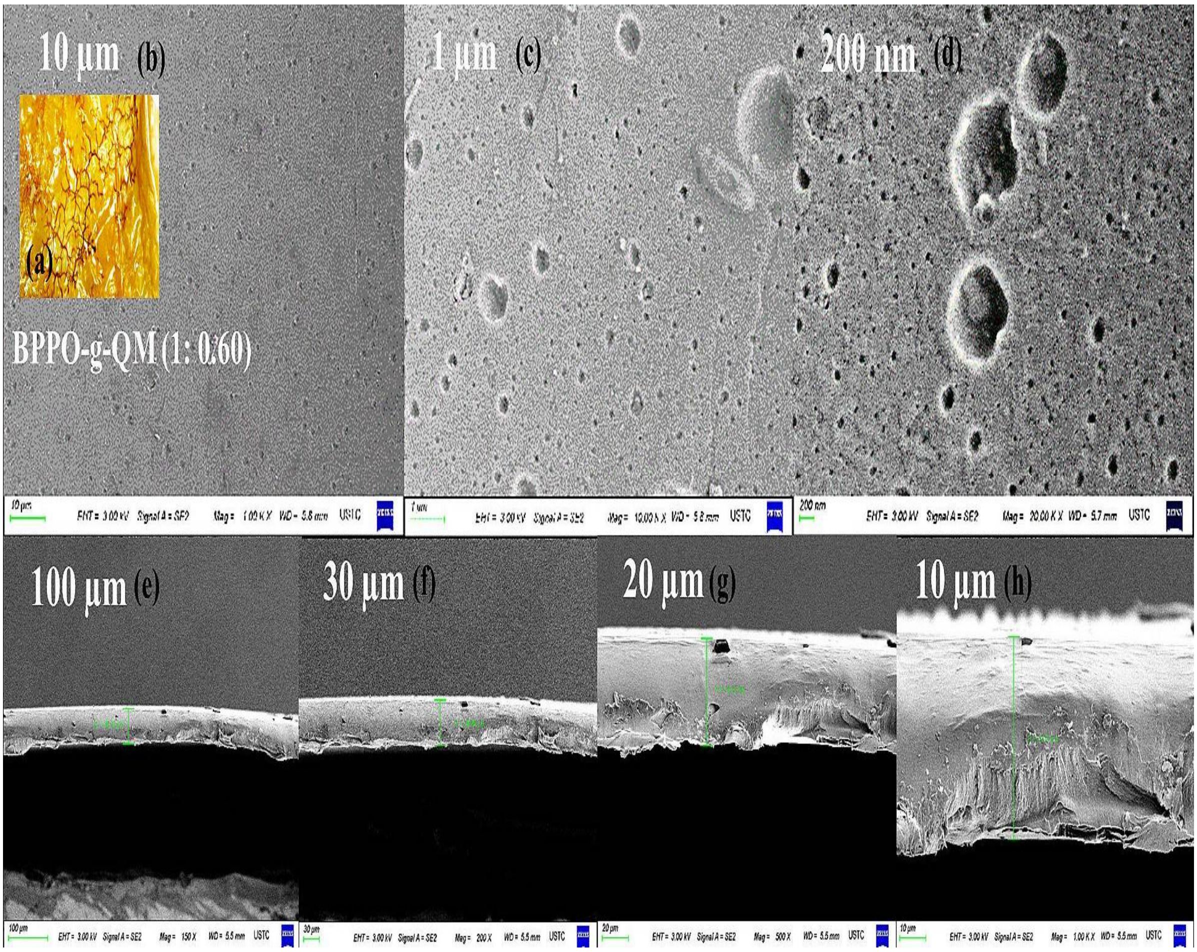


**Figure S11.** (a) Prepared BPPO-g-QM (1:0.60) membrane and Field Emission Scanning Electron Microscope (FE-SEM, Sirion500, FEI Company, USA) as consisting magnification of: (b) 10 µm (c) 1 µm and (d) 200 nm sequentially & Cross-section regarding and (e) 100 µm respectively.

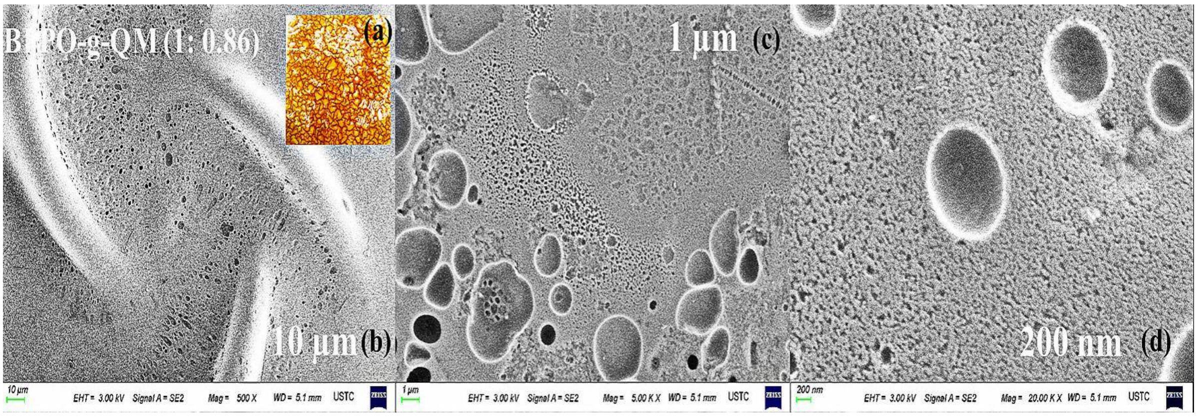


**Figure S12.** (a) Prepared BPPO-g-QM (1:0.86) membrane and Field Emission Scanning Electron Microscope (FE-SEM, Sirion500, FEI Company, USA) as consisting magnification of: (b) 10 µm (c) 1 µm, and (d) 200 nm respectively.

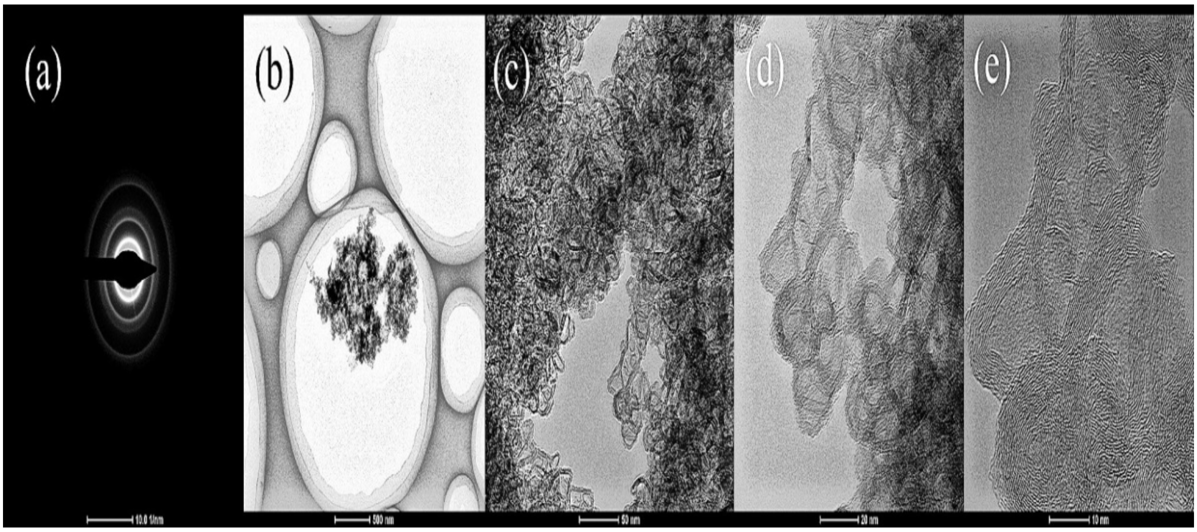


**Figure S13.** Field Emission High Resolution Transmission Electron Microscope (FE-HRTEM, FEI, Talos F200X, USA) as low dose (a) conformation test & as consisting magnification of: (b) 500 nm (c) 50 nm (d) 20 nm, and (e) 10 nm respectively.

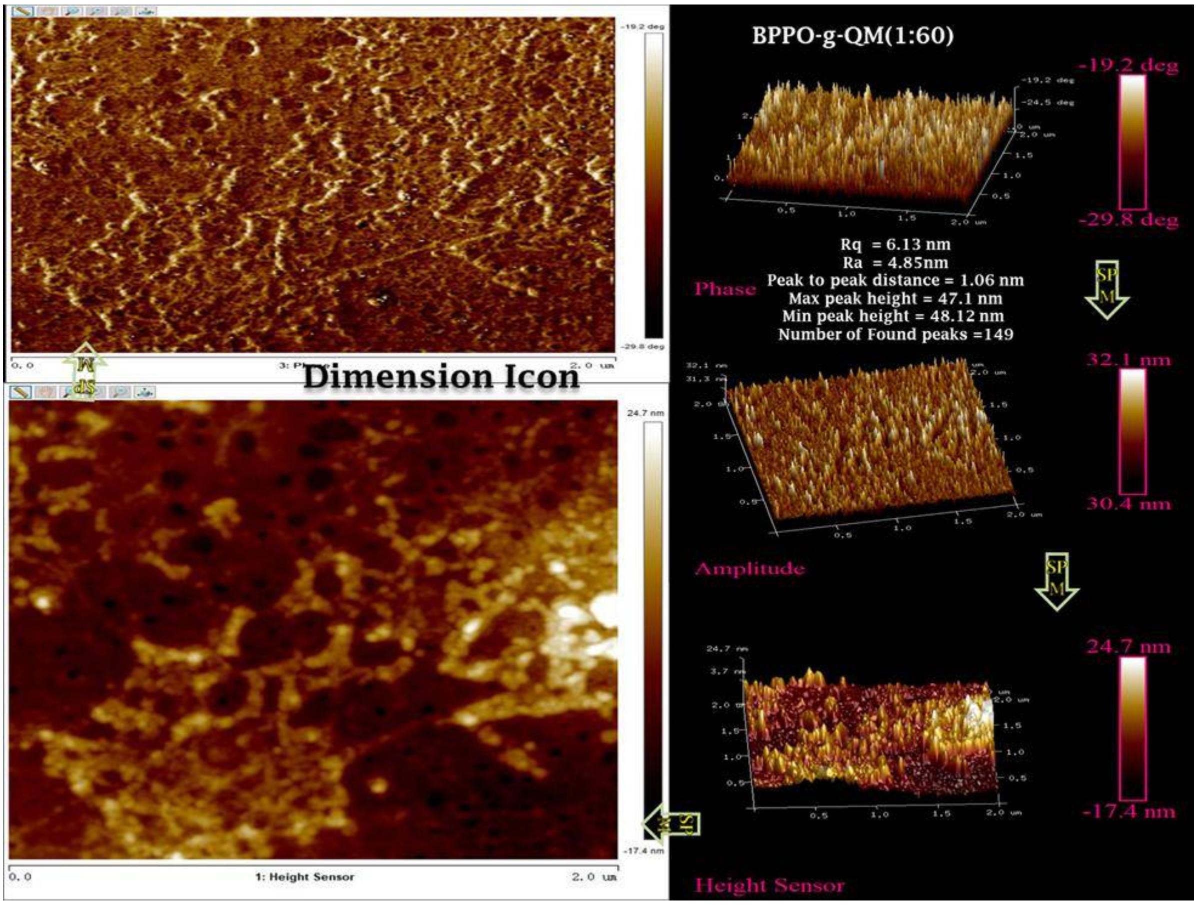


**Figure S14.** Scanning Probe Microscopy as AFM of tiny images of the surface as regarding the Rq and Ra value are 6.13 nm and 4.85 nm respectively; Moreover peak to peak distance approximately 1.06 nm and number of found peak 149 as well.

## References

1. Xu, T.; Yang, W. Fundamental studies of a new series of anion exchange membranes: membrane preparation and characterization.*J. Membr. Sci*. **2001**, *190*, 159-166.
2. Hossain, M.M., Hou, J.; Wu, L.; Ge, Q.; Liang, X.; Mondal, A.N.; Xu, T. Anion exchange membranes with clusters of alkyl ammonium group for mitigating water swelling but not ionic conductivity. *J. Membr. Sci*. **2018**, *550*, 101-109.
3. Xu, T.; Wu, D.; Wu, L. Poly (2, 6-dimethyl-1, 4-phenylene oxide)(PPO)—a versatile starting polymer for proton conductive membranes (PCMs). *Progr. Polym. Sci*. **2008**, *33*, 894-915.
4. Dang, H.-S.; Jannasch, P. Exploring different cationic alkyl side chain designs for enhanced alkaline stability and hydroxide ion conductivity of anion-exchange membranes. *Macromol*. **2015**, *48*, 5742-5751.
5. Fane, A.G.; Wang, R.; Jia, Y. Membrane and Desalination Technologies in *Handbook of Environmental Engineering*, Wang, L.K. Chen J.; Hung Y.; Shammas N.K. Humana Press: Totowa, United States, **2011**; pp. 3-45.
6. Ran, J., Wu, L.; Ge, Q.; Chen, Y.; Xu, T. High performance anion exchange membranes obtained through graft architecture and rational cross-linking. *J. Membr. Sci*. **2014**, *470*, 229-236.
7. Kariduraganavar, M.Y., et al., Ion-exchange membranes: preparative methods for electrodialysis and fuel cell applications. *Desalination.* **2006**, 197, 225-246.
8. Liu, Y., Pan, L.; Xu, X.; Lu, T.; Sun, Z. Chua D.H.C. Enhanced desalination efficiency in modified membrane capacitive deionization by introducing ion-exchange polymers in carbon nanotubes electrodes. *Electrochim. Acta*. **2014**, *130*, 619-624.
9. Liu, X., Li, Q.; Jiang, C.; Lin, X.; Xu, T. Bipolar membrane electrodialysis in aqua–ethanol medium: Production of salicylic acid. *J. Membr. Sci*. **2015**, *482*, 76-82.
10. Ge, L., Wu, B.; Li, Q.; Wang, Y.; Yu, D.; Wu, L.; Pan, J.; Miao, J.; Xu, T. Electrodialysis with nanofiltration membrane (EDNF) for high-efficiency cations fractionation. *J. Membr. Sci*. **2016**, *498*, 192-200.
11. Luo, J., Wu, C.; Wu, Y.; Xu, T. Diffusion dialysis of hydrochloride acid at different temperatures using PPO–$SiO_2$ hybrid anion exchange membranes. *J. Membr. Sci*. **2010**, *347*, 240-249.

© 2020 by the authors. Submitted for possible open access publication under the terms and conditions of the Creative Commons Attribution (CC BY) license (http://creativecommons.org/licenses/by/4.0/).
